# Supplementary figures and images for: Dietary flavonoids may improve insulin resistance: NHANES, network pharmacological analyses and in vitro experiments
Source: PLoS One. 2025 Dec 5;20(12):e0338100. doi: 10.1371/journal.pone.0338100 (PMC12680246; doi:10.1371/journal.pone.0338100)

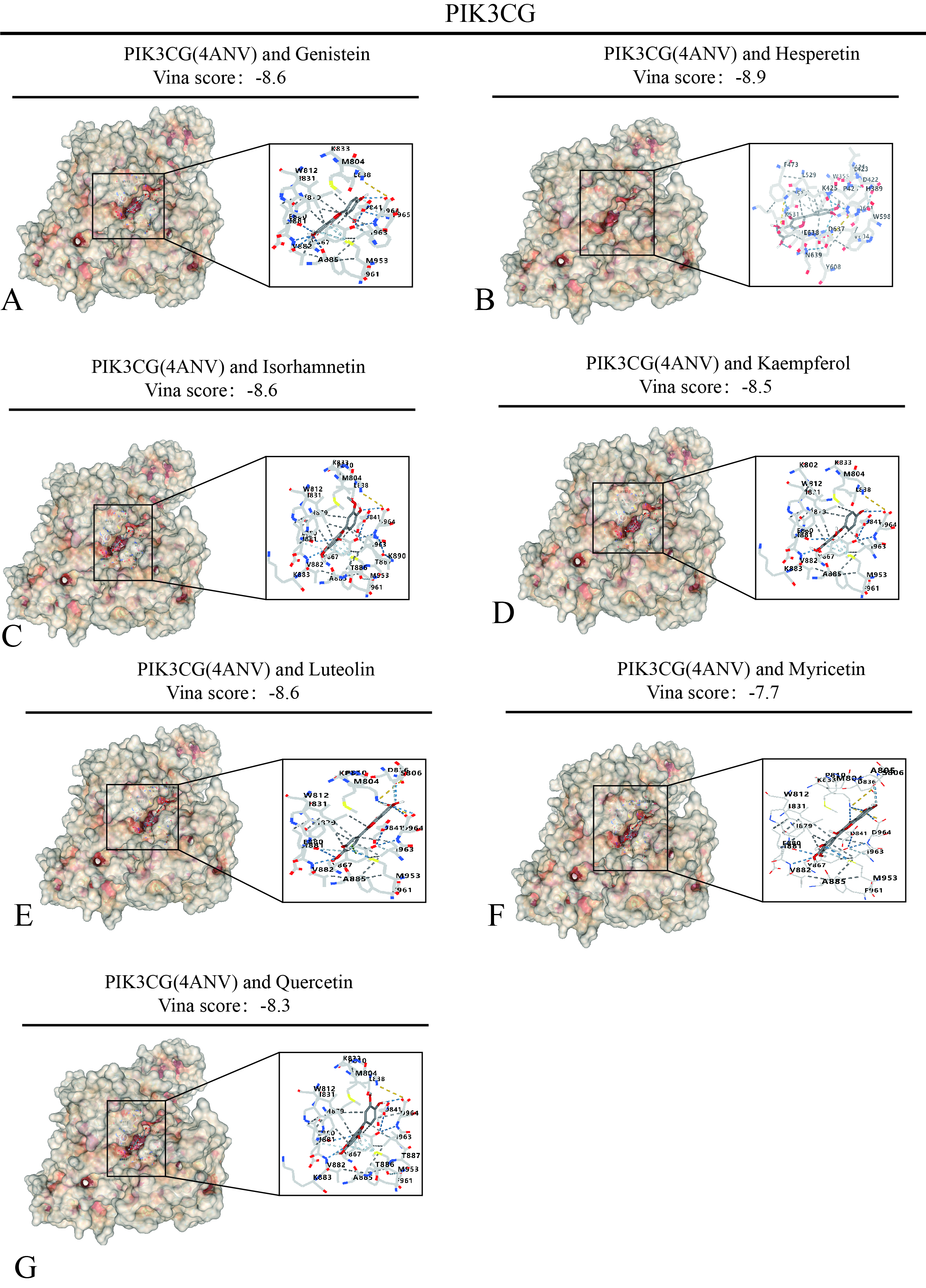

Supplement: S1 Fig — (TIFF) [file pone.0338100.s001.tiff]

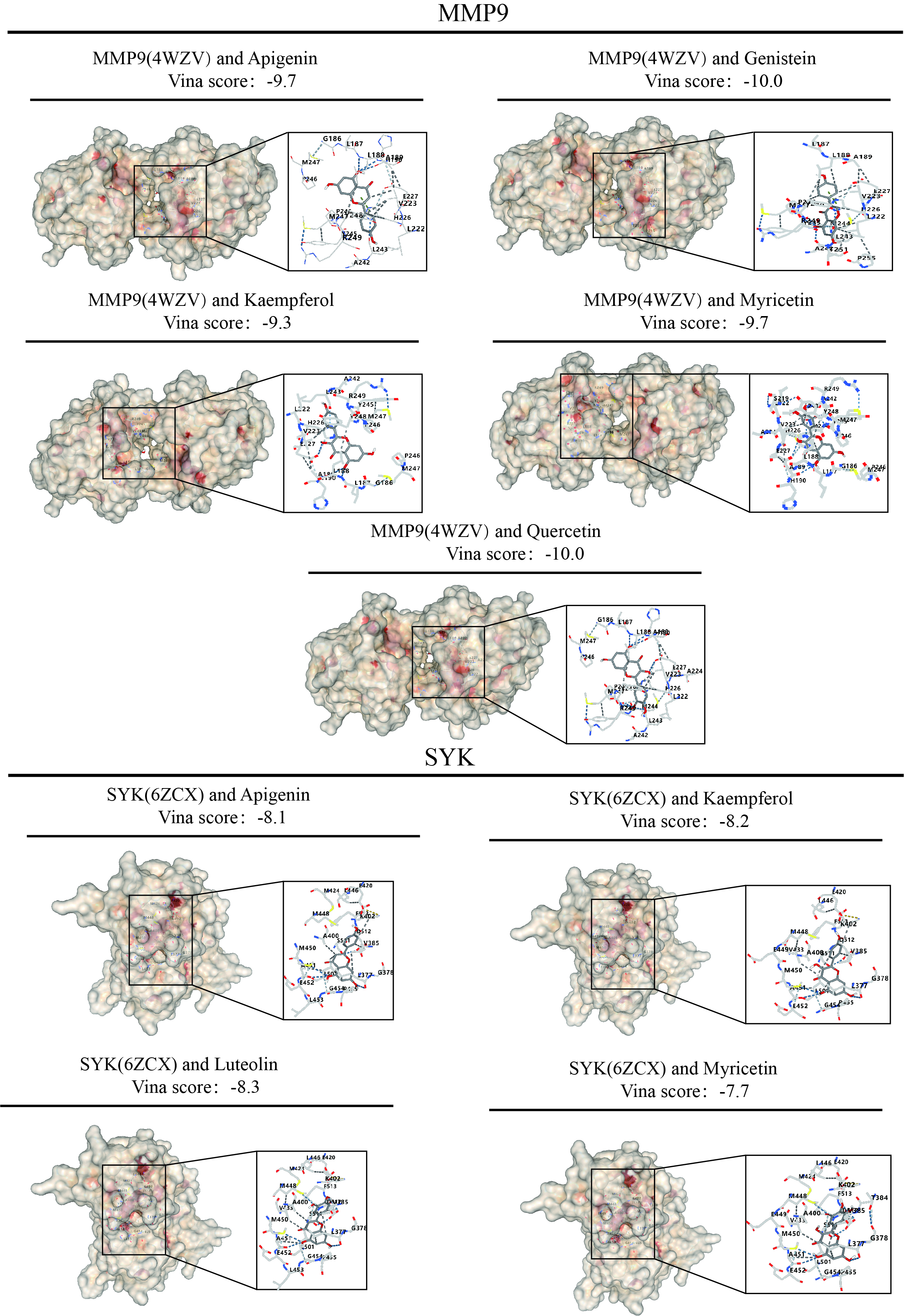

Supplement: S2 Fig — (TIFF) [file pone.0338100.s002.tiff]

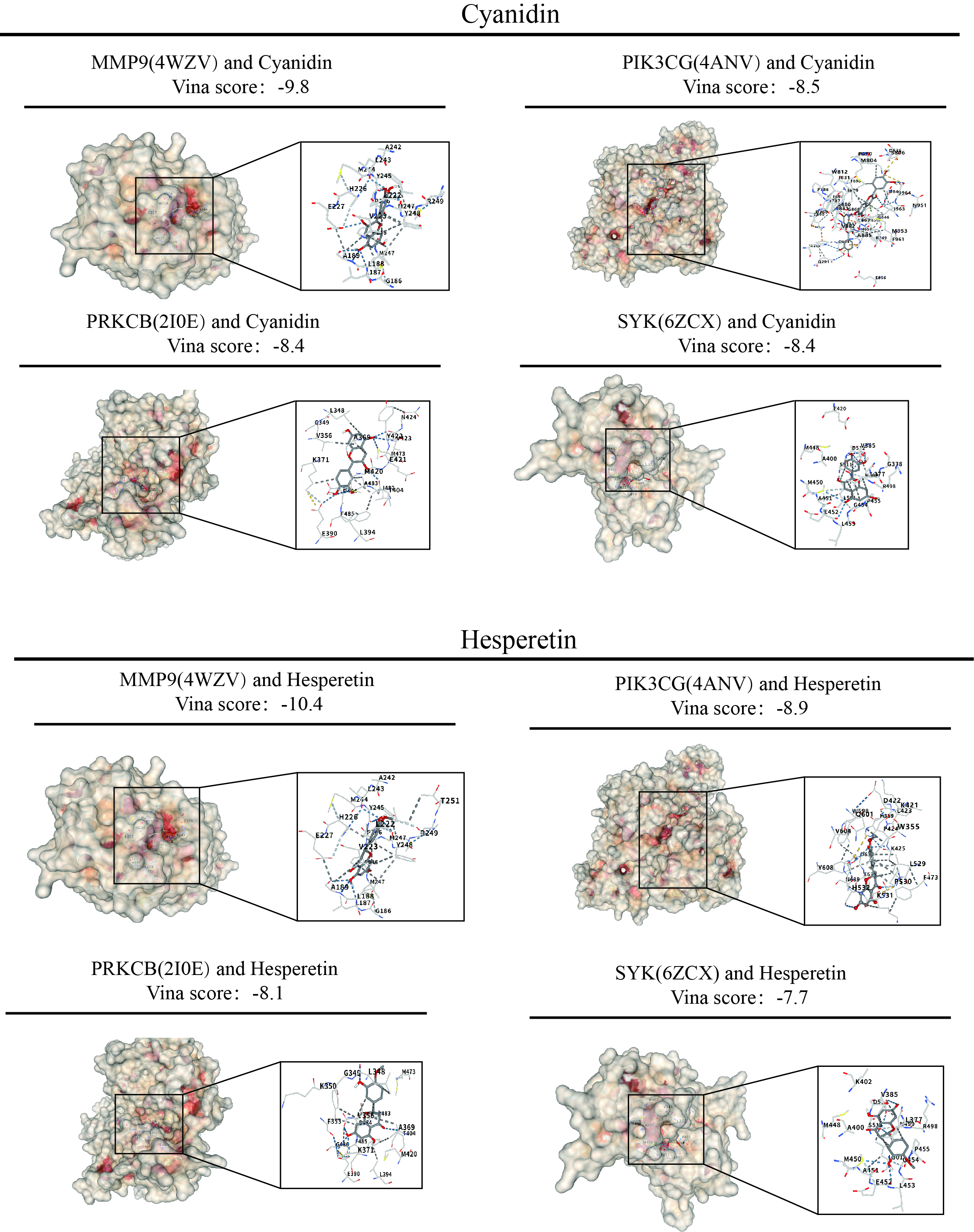

Supplement: S3 Fig — (TIFF) [file pone.0338100.s003.tiff]

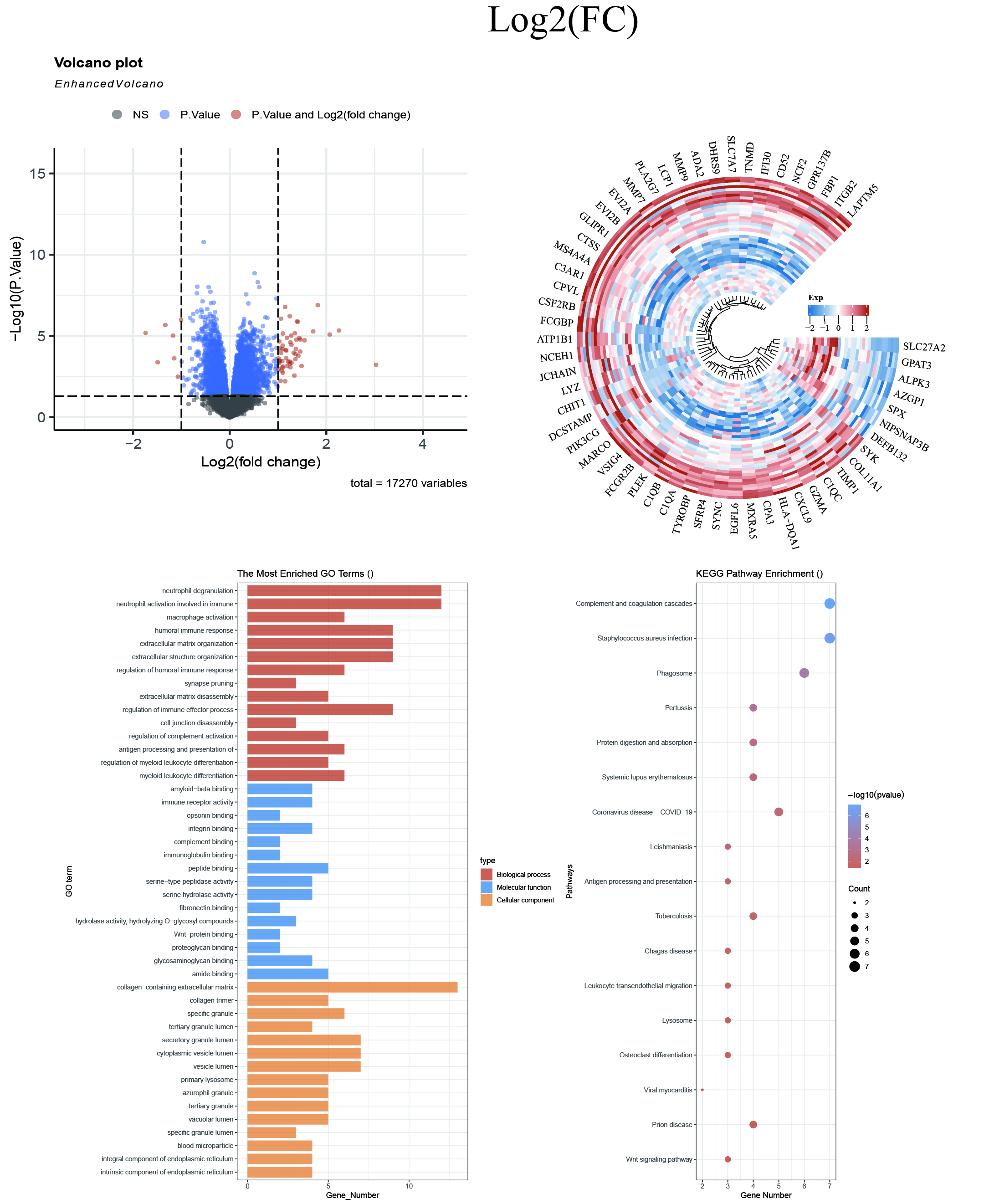

Supplement: S4 Fig — (TIFF) [file pone.0338100.s004.tiff]
