# Supplementary material for: Dietary flavonoids may improve insulin resistance: NHANES, network pharmacological analyses and in vitro experiments
Source: PLoS One. 2025 Dec 5;20(12):e0338100. doi: 10.1371/journal.pone.0338100 (PMC12680246; doi:10.1371/journal.pone.0338100)
Supplement: S3 Table — Adjusted by age, sex, race, education, PIR, smoking status, drinking status, caloric intake, physical activity, coronary heart disease, and hypertension. Total Sum of all 29 flavonoids: [0,50]mg/d, (50,200]mg/d, (200,500]mg/d, > 500mg/d. Total Anthocyanidins: [0,0.15]mg/d, (0.15,2.36]mg/d, (2.36,13.6]mg/d, > 13.6mg/d. Total Isoflavones: [0,0]mg/d, (0.001,0.015]mg/d, (0.02,0.1]mg/d, > 0.1mg/d. Total Flavan_3_ols: [0,5.3]mg/d, (5.03,17]mg/d, (17,170] mg/d, > 170mg/d. Total Flavanones: [0,0.1]mg/d, (0.1,1]mg/d, (1,20]mg/d, > 20mg/d. Total Flavones: [0,0.2]mg/d, (0.2,0.6]mg/d, (0.6,1.2]mg/d, > 1.2mg/d. Total Flavonols: [0,8]mg/d, (8,14]mg/d, (14,24]mg/d, > 24mg/d. (DOCX) [file pone.0338100.s007.docx]

| **Character** | **Very low** | **Low** | | **Moderate** | | **High** | | **P for interaction** |
| --- | --- | --- | --- | --- | --- | --- | --- | --- |
|  |  | **β (95% CI)** | **P-value** | **β (95% CI)** | **P-value** | **β (95% CI)** | **P-value** |  |
| **Total Isoflavones (mg/d)** |  |  |  |  |  |  |  | 0.24 |
| Very low | ref | -3.28(-5.66,-0.90) | 0.01 | -2.79(-4.53,-1.05) | 0.003 | -0.34(-2.45, 1.77) | 0.74 |  |
| Low | ref | -1.53( -5.41, 2.35) | 0.42 | -3.21( -6.71, 0.29) | 0.07 | -1.48( -5.57, 2.61) | 0.46 |  |
| Moderate | ref | -1.68( -4.52, 1.16) | 0.23 | -0.09( -3.53, 3.34) | 0.96 | -1.56( -4.80, 1.69) | 0.33 |  |
| High | ref | -0.95(-4.44, 2.55) | 0.58 | -1.43(-5.03, 2.18) | 0.42 | -2.1(-5.96, 1.75) | 0.27 |  |
| **Total Anthocyanidins (mg/d)** |  |  |  |  |  |  |  | 0.29 |
| Very low | ref | -2.07(-5.61, 1.47) | 0.24 | -1.71(-4.81, 1.38) | 0.26 | 0.67(-1.70, 3.05) | 0.56 |  |
| Low | ref | 0.43( -2.03, 2.90) | 0.72 | 0.40( -2.17, 2.97) | 0.75 | 0.20( -2.84, 3.24) | 0.89 |  |
| Moderate | ref | -3.09( -6.21, 0.02) | 0.05 | -1.39( -4.27, 1.49) | 0.33 | -2.80( -5.87, 0.27) | 0.07 |  |
| High | ref | 1.19( -9.14,11.53) | 0.81 | 0.98( -9.61,11.58) | 0.85 | 1.45( -8.86,11.75) | 0.77 |  |
| **Total Flavan-3-ols (mg/d)** |  |  |  |  |  |  |  | 0.07 |
| Very low | ref | -2.18( -4.86,0.49) | 0.11 | -1.24( -4.64,2.15) | 0.46 | -2.76(-13.23,7.71) | 0.59 |  |
| Low | ref | -2.32( -4.68, 0.04) | 0.05 | -1.30( -4.26, 1.66) | 0.37 | 1.37(-11.66,14.40) | 0.83 |  |
| Moderate | ref | -2.12(-15.08,10.84) | 0.74 | -3.79(-16.39, 8.80) | 0.54 | -8.64(-21.78, 4.49) | 0.19 |  |
| High |  | ref |  | -0.06(-5.87, 5.75) | 0.98 | 1.86(-3.83, 7.55) | 0.51 |  |
| **Total Flavanones (mg/d)** |  |  |  |  |  |  |  | 0.28 |
| Very low | ref | -1.23(-4.03, 1.57) | 0.37 | -3.78(-7.18,-0.37) | 0.03 | -0.82(-3.52, 1.87) | 0.53 |  |
| Low | ref | -0.7(-3.74,2.34) | 0.64 | 0.38(-2.53,3.29) | 0.79 | 0.05(-2.34,2.44) | 0.97 |  |
| Moderate | ref | -2.45(-5.15, 0.24) | 0.07 | -3.56(-6.26,-0.86) | 0.01 | -1.44(-4.70, 1.82) | 0.37 |  |
| High | ref | 7.59( -3.91,19.10) | 0.18 | 10.25( -0.86,21.36) | 0.07 | 9.00( -2.68,20.68) | 0.12 |  |
| **Total Flavones (mg/d)** |  |  |  |  |  |  |  | 0.04 |
| Very low | ref | -1.51(-4.44,1.42) | 0.3 | -2.53(-5.24,0.18) | 0.07 | 4.6( 0.62,8.57) | 0.03 |  |
| Low | ref | -2.43( -5.24, 0.38) | 0.09 | -1.33( -4.25, 1.58) | 0.35 | -1.49( -4.45, 1.46) | 0.31 |  |
| Moderate | ref | -4.38(-7.90,-0.87) | 0.02 | -2.35(-5.17, 0.47) | 0.1 | -3.01(-5.58,-0.43) | 0.02 |  |
| High | ref | -1.72(-6.59, 3.14) | 0.47 | -3.39(-7.80, 1.03) | 0.13 | -2.59(-7.91, 2.72) | 0.32 |  |
| **Total Flavonols (mg/d)** |  |  |  |  |  |  |  | 0.72 |
| Very low | ref | -2.65( -5.13, -0.17) | 0.04 | -2.43( -5.52, 0.67) | 0.12 | -17.63(-23.33,-11.93) | <0.0001 |  |
| Low | ref | -0.86(-2.61, 0.89) | 0.32 | -0.72(-3.23, 1.79) | 0.56 | -0.45(-5.91, 5.02) | 0.87 |  |
| Moderate | ref | -2.51( -6.86, 1.85) | 0.25 | -3.41( -7.56, 0.74) | 0.1 | -1.8( -5.94, 2.34) | 0.38 |  |
| High | ref | 2.03(-1.97,6.03) | 0.3 | 0.74(-3.82,5.31) | 0.74 | 3.15(-1.05,7.35) | 0.13 |  |
