# Supplementary material for: Dietary flavonoids may improve insulin resistance: NHANES, network pharmacological analyses and in vitro experiments
Source: PLoS One. 2025 Dec 5;20(12):e0338100. doi: 10.1371/journal.pone.0338100 (PMC12680246; doi:10.1371/journal.pone.0338100)
Supplement: S4 Table — (DOCX) [file pone.0338100.s008.docx]

**Table S4** List of 29 types of flavonoid compounds

| **Flavonoid class** | **Flavonoid** | **Inclusion** | **Reasons for Exclusion** |
| --- | --- | --- | --- |
| Anthocyanidins | Cyanidin | Yes. |  |
| Anthocyanidins | Delphinidin | Yes. |  |
| Anthocyanidins | Malvidin | No. | No target was found within the threshold range. |
| Anthocyanidins | Pelargonidin | No. | No target was found within the threshold range. |
| Anthocyanidins | Peonidin | No. | No target was found within the threshold range. |
| Anthocyanidins | Petunidin | No. | No target was found within the threshold range. |
| Flavan-3-ols | (-)-Epicatechin* | No. | No target was found within the threshold range. |
| Flavan-3-ols | (-)-Epicatechin 3-gallate* | No. | No target was found within the threshold range. |
| Flavan-3-ols | (-)-Epigallocatechin* | No. | No target was found within the threshold range. |
| Flavan-3-ols | (-)-Epigallocatechin 3-gallate* | No. | No target was found within the threshold range. |
| Flavan-3-ols | (+)-Catechin* | No. | No target was found within the threshold range. |
| Flavan-3-ols | (+)-Gallocatechin* | Yes. |  |
| Flavan-3-ols | Theaflavin | No. | No target was found within the threshold range. |
| Flavan-3-ols | Theaflavin-3,3'-digallate | No. | No target was found within the threshold range. |
| Flavan-3-ols | Theaflavin-3'-gallate | No. | No target was found within the threshold range. |
| Flavan-3-ols | Theaflavin-3-gallate | No. | No target was found within the threshold range. |
| Flavan-3-ols | Thearubigins | No. | No target was found within the threshold range. |
| Flavanones | Eriodictyol | Yes. |  |
| Flavanones | Hesperetin | Yes. |  |
| Flavanones | Naringenin | No. | No target was found within the threshold range. |
| Flavones | Apigenin | Yes. |  |
| Flavones | Luteolin | Yes. |  |
| Flavonols | Isorhamnetin | Yes. |  |
| Flavonols | Kaempferol | Yes. |  |
| Flavonols | Myricetin | Yes. |  |
| Flavonols | Quercetin | Yes. |  |
| Isoflavones | Daidzein | No. | No target was found within the threshold range. |
| Isoflavones | Genistein | Yes. |  |
| Isoflavones | Glycitein | No. | No target was found within the threshold range. |
