# Supplementary material for: Dietary flavonoids may improve insulin resistance: NHANES, network pharmacological analyses and in vitro experiments
Source: PLoS One. 2025 Dec 5;20(12):e0338100. doi: 10.1371/journal.pone.0338100 (PMC12680246; doi:10.1371/journal.pone.0338100)
Supplement: Attachment — Submitted filename: Reviewer.pdf [file pone.0338100.s012.pdf]

Minor

The revised manuscript has been improved, and just minor aspects should be checked.

- Material and methods: Section 2.7: Please, state clearly the number of cells seeded to perform each assay.
  For the RT-qPCR, please, also include the micrograms of RNA transcribed and the nanograms of RNA amplified.

There are still certain grammatical and typo errors through the manuscript that require attention.
