# Supplementary material for: Dietary flavonoids may improve insulin resistance: NHANES, network pharmacological analyses and in vitro experiments
Source: PLoS One. 2025 Dec 5;20(12):e0338100. doi: 10.1371/journal.pone.0338100 (PMC12680246; doi:10.1371/journal.pone.0338100)
Supplement: Attachment — Submitted filename: Response to Reviewers.docx [file pone.0338100.s011.docx]

Dear editor of the *PLOS ONE*,

We are submitting our revised manuscript entitled “Dietary flavonoids may improve insulin resistance: NHANES, network pharmacological analyses and in vitro experiments” to the *PLOS ONE* for your reconsideration of its suitability for publication. All authors have read and approved the manuscript. We have carefully taken the reviewers’ comments into account and provided responses to each of the points raised by the reviewers. Please refer to the present manuscript text.

**Reviewer #1**

1.The manuscript evaluates the connection between dietary flavonoid intake and insulin resistance (IR) by combining NHANES database and network pharmacology, to later investigate the effect of two polyphenols on cell viability and glucose uptake in adipocytes. A major concern arises from the fact that there seems to be a disconnection among results. Thus, cell culture approach seems to be based on the results obtained from the NHANES study. Then, what is the purpose of the pharmacological network approach? Please, note that polyphenols are not drugs.

2.Title: it seems that the authors have focussed on obesity, although IR is present in other diseases. If this is the case, this should be included in the title, and if not, consider other diseases (diabetes, metabolic syndrome, etc.).

3.Introduction: This section is focused on obesity and insulin resistance, but IR is also and importantly present in diabetes. Please, consider.

4.Section 3.1: Insulin levels have not been included in the baseline characteristics (Table 1). Would it possible to include this data? Please, explain.

5.Section 3.7: Line 304, was just the fat of the patients assayed for these genes? Please, explain.

6.Section 3.8: how was the interaction of the selected polyphenols for the cell culture studies with the identifies key proteins?

7.Section 3.9: It is not clear the criteria for selecting anthocyanidins and hesperidin to perform the in vitro studies. What is their connection with the previous results showed in the manuscript? Which was the purpose of performing the target acquisition and the docking approaches?

8.Regarding the anthocyanidins, was this a mixture of compounds? Please, explain.

9.Please, note that the glucose uptake was assayed instead of the glucose intake.

10.According to GO and KEGG analysis, inflammation, fatty acid metabolism, insulin and AMPKs pathways were considered the main targets of flavonoids. Why were not any of these aspects assayed? In this regard, it would be desirable an additional demonstration of the polyphenol-regulated effects at least on the inflammation by analysing proteins levels of any selected remarkable modulated one related to this pathway, such as PIK3CG (key protein that authors have identified to interact with different polyphenol), TNF-alpha, NF-kB, etc.

11. Discussion: Line 426, what do the authors mean “…we synthesized all 12 dietary flavonoid genes”? Please, rephrase the sentence (genes identified?).

12.How realistic are the doses used in the cell culture analysis? Could ever adipocytes be exposed to those concentrations? Could these doses be achieved through the diet? Is anything known about the bioavailability of these compounds? Please, explain and discuss the relevance of the concentrations used.

13.Tables: Please, note that legends of tables should be placed before the table.

14.Figures: Text in figure 3 is not readable. Please, try to improve that.

15.For consistency, in figure 4, please, place the subheadings (A, B, C and so on) at the top of each figure.

16.Please, provide the complete name before using an abbreviation (METS-IR, FGF, TNF-alpha, UCP-1 etc.)

**To Reviewer #1**

1. Thank you very much for your hard work. It has been very helpful for improving the quality of our articles. To address the reviewer's concerns, we emphasize that the integration of network pharmacology in this study serves to elucidate the multi-target mechanisms by which dietary flavonoids ameliorate IR, ensuring methodological coherence across our tripartite approach:
(1) NHANES analyses identified anthocyanidins and flavanones as flavonoid subclasses significantly inversely associated with IR (Table 2);
(2) network pharmacology—a well-established methodology for studying bioactive natural compounds (Refs. 35, 38, 52)—predicted their core targets (e.g., PIK3CG, MMP9) and enriched pathways (e.g., NF-κB, AMPK), providing mechanistic hypotheses for experimental validation (Figs. 5-6);
(3) in vitro experiments confirmed these predictions, demonstrating restored glucose uptake and mitochondrial function (Fig. 7).

While flavonoids are dietary components, the term "drug-like effects" denotes their ability to modulate specific targets (e.g., suppressing PIK3CG-mediated inflammation) and aligns with standard practices in nutraceutical research. Molecular docking (Vina Score<−5kcal/mol) further validated strong binding affinities, bridging epidemiological observations with mechanistic insights. We are open to revising terminology if "drug-like" causes confusion, but the methodology remains valid for deciphering multi-target actions of bioactive food compounds. This strategy avoids arbitrary in vitro testing and ensures logical continuity from population data to cellular mechanisms.

2. Thank you very much for your valuable suggestions on this article. We fully understand and agree with your view that IR is a core mechanism involving multiple pathological states. This article does indeed overlook other diseases that may lead to IR (T2DM, MetS, etc.). After carefully considering your opinions, we did not modify the title but made the following changes to more comprehensively reflect the wide correlation of IR and enhance the universality of the research:
(1) First, we revised the introduction content, clearly expounds the IR is not only the key pathological link in obesity, T2DM, MetS, CVD, NAFLD, PCOS, and some of many diseases such as neurodegenerative diseases important driving factor. (lines 29-67).
(2) Second, in using NHANES data analysis as well as the network pharmacology gene screening differences, we are not confined to obese people will study object. The NHANES analysis included a broad adult population (including both obese and non-obese individuals) who met the criteria and had complete IR assessment data. Network pharmacological analysis IS also based on a broader dataset of IR-related gene expression, which includes adipose tissue samples of IR and insulin-sensitive (IS), and the source population does not specifically refer to obese individuals.
(3) Finally, part of the discussion, we complement and strengthen on the mechanism of action of dietary flavonoids improve IR may also apply to hyperlipidemia, and metabolic dysfunction related to fatty liver disease and other IR related metabolic disease (lines 424-432). We explored the anti-inflammatory and antioxidant properties of flavonoids and their regulatory effects on key signaling pathways, which have universality beyond specific causes (such as simple obesity).

These modifications establish that the effect of dietary flavonoids in improving IR is pleiotropic, and its potential benefits are relatively independent of the specific primary disease in which IR occurs.

3. We sincerely appreciate your emphasis on the broad spectrum of diseases related to IR and fully agree that IR is not limited to obese individuals. To address this key point, we revised the introduction section and fundamentally reconstructed the paper, clearly stating that IR is not only a key pathological link in obesity but also an important driving factor for numerous diseases such as T2DM, MetS, CVD, NAFLD, PCOS, and certain neurodegenerative diseases. These changes ensure that the introduction accurately reflects the prevalence of IR in various metabolic diseases, especially diabetes, in line with the valuable insights of the reviewers, providing a more comprehensive basis for our research. For specific revision details, please refer to lines 29 to 67 of the manuscript.
4. Thank you for your important suggestions. Regarding the suggestions for supplementing insulin level data in the baseline characteristics table (Table 1), we explain as follows: Firstly, the gold standard for evaluating IR is the hyperinsulin-normal blood glucose clamp test. However, due to the complexity of the equipment, the strong invasiveness of the operation and the difficulty in clinical implementation of this method, it is difficult to be widely applied in large-scale epidemiological studies. Therefore, based on the objective limitations of the NHANES database, metabolic insulin resistance index (METS-IR) was selected as an alternative marker for IR in this study. The calculation formula is: METS-IR = Ln[(2 × fasting blood glucose) + fasting triglycerides] × body mass index/Ln(high-density lipoprotein cholesterol). The advantage of this index lies in the fact that it only requires conventional metabolic parameters (without the need to directly measure insulin levels), and it has been verified to be significantly correlated with the gold standard results. Although METS-IR itself does not depend on insulin levels, we fully understand your requirements for data integrity. Fasting insulin level data have been added to Table 1 of the revised draft to provide a more comprehensive baseline characteristic description. Thank you again for your guidance on the rigor of the research.

| **Variable** | **Total (n=3564)** | **Very low (n=1458)** | **Low**  **(n=1115)** | **Moderate (n=568)** | **High**  **(n=423)** | **P-value** |
| --- | --- | --- | --- | --- | --- | --- |
| **Fasting insulin (pmol/L)** | 12.01±0.29 | 12.19±0.35 | 12.13±0.39 | 11.00±0.53 | 12.42±0.83 | 0.07 |

5. We selected the GEO database datasets GSE20950 and GSE26637 for the subsequent study. The detection content of these two datasets was the transcriptome sequencing of adipose tissue from patients with insulin resistance and insulin-sensitive individuals, rather than that from patients with insulin resistance alone. The reason for choosing adipose tissue is that it is a core participant in IR. It can store excess lipids, secrete adipokines and drive inflammation through the recruitment of immune cells.
6. Thank you very much for your suggestion. We present the interaction between the dietary flavonoids selected in the experiment and the key proteins in Figure S3. This result indicates that there is a good possibility of interaction between Cyanidin and Hesperetin and the key proteins.
7. The selection of anthocyanidins and hesperidin for in vitro studies was based on the significant negative associations observed in the NHANES analysis and their established biological relevance in improving IR. Specifically, anthocyanidins exhibited the strongest inverse correlation with METS-IR (P<0.0001), while flavanones (represented by hesperidin, a prominent citrus-derived compound) also showed a significant association (P<0.001). These findings were further supported by nonlinear dose-response analyses, which highlighted their potential therapeutic thresholds. Additionally, network pharmacology identified key targets (e.g., PIK3CG, MMP9) shared by these flavonoids, linking them to IR-related pathways such as inflammation and mitochondrial function. The in vitro experiments confirmed their efficacy by demonstrating restored glucose uptake and improved mitochondrial membrane potential in IR-induced adipocytes, aligning with the mechanistic insights from the computational analyses. Thus, the choice of anthocyanidins and hesperidin was grounded in their statistical significance, biological plausibility, and consistency with the study’s multi-level evidence.

The purpose of performing target acquisition and molecular docking approaches in this study was to identify and validate the potential molecular mechanisms through which dietary flavonoids improve IR. By integrating network pharmacology and molecular docking, we aimed to pinpoint key genes and pathways involved in IR that interact with flavonoids. Target acquisition involved screening differential genes from IR and insulin-sensitive adipose tissue datasets, followed by identifying overlapping targets between these genes and flavonoid-related targets. Molecular docking was then used to evaluate the binding affinity between specific flavonoids and hub genes (e.g., PIK3CG, MMP9, SYK, PRKCB), confirming their potential interactions. These approaches provided mechanistic insights, revealing that flavonoids may ameliorate IR by modulating inflammatory responses, mitochondrial function, and signaling pathways such as NF-κB and PI3K-Akt, thus bridging the gap between epidemiological findings and molecular-level evidence.

8. Based on the analysis of the NHANES database, initially we selected Cyanidin to represent anthocyanidins in the diet for the experiment, because Cyanidin is the most common among anthocyanidins. However, since the free form compound did not exist, we conducted subsequent experiments using the stable salt form Cyanidin Chloride(HY-N0499,MCE) with the same biological activity.
9. Thank you very much for your suggestion. It has corrected our long-standing translation mistakes. We have already corrected the relevant content. Thank you again for your hard work.
10. We sincerely appreciate your valuable suggestions, which have been tremendously helpful for improving our manuscript. To further validate the effects of flavonoids on PIK3CG and TNF-α, we employed RT-qPCR to analyze the impact of five dietary flavonoids on PIK3CG expression levels in 3T3-L1-derived adipocytes. Additionally, we utilized ELISA to measure TNF-α concentrations in the culture supernatants of these treated adipocytes (Fig. 7D-E).
11. Thank you very much for your suggestion. There are indeed significant issues with the expression in the original text. We have changed it to “Through integrated analysis of all insulin resistance-related differentially expressed genes and their. intersection with 12 dietary flavonoid-associated genes, we identified 5 overlapping key genes.”
12. Thank you very much for your suggestion. We have incorporated relevant content into the discussion, which has made the article more readable. (Lines 557-577)
13. Thank you for your valuable suggestions. We have strictly followed academic norms to modify the full text table: Firstly, it has been uniformly changed to a three-line table format. At the same time, adjust the title positions and move all titles to the top of the table. Finally, ensure that the modified table fully complies with the requirements of the journal and that no original data has been altered.
14. Thank you very much for your meticulous review of our manuscript and the valuable suggestions you have provided! Your feedback is crucial for enhancing the clarity and presentation quality of our research. We fully understand the problem you pointed out that the text in Figure 3 is difficult to read. We have taken effective measures for optimization, splitting Figure 3 (A-H) into Figure 3 (A-D) and Figure 4 (A-D), and ensuring that all text elements in the charts are clearly distinguishable.
15. Thank you for pointing out the issue that the placement of the subheadings (A, B, C, etc.) in Figure 4 is inconsistent with that of other charts. We agree that maintaining the uniformity of chart formats is of great significance for enhancing the professionalism and readability of manuscripts. Regarding the issue of the subtitle position in Figure 4, we have, in accordance with your requirements (and consistent with other charts), uniformly placed the subtitles (A, B, C, etc.) at the top of each subgraph.
16. We are very grateful to you for pointing out this important normative issue. You are completely correct - we were negligent in the rigor of the expression of terms and failed to follow the academic principle of "using the full name for the first appearance and marking the abbreviation". We sincerely apologize for this and have marked the full names and abbreviations of all terms when they first appear in the full text. This revision has covered all chapters of the manuscript, including the main text, tables and tables, and appendices. Meanwhile, researchers whose native language is English will conduct the final language verification of the manuscript content. We once again apologize for the previous non-standard expression and thank you for helping us enhance the professional rigor of the paper.

**Reviewer #2**

1.The manuscript contains multiple formatting issues that require thorough revision by the authorss Specific instances include the presence of Chinese characters and excessive spacing throughout the text.

2. The fundamental methodological approach to quartile analysis is flawed, as divisions should reflect compound concentration gradients rather than equal population proportions. This represents the most serious methodological flaw in the manuscript.

3. The dataset covers the periods 2007–2010 and 2017–2018. Could the authors clarify why no data from the intervening years were included in the analysis?

4. Several figures (e.g., Fig.1, Fig. 2) lack sufficient resolution, which may hinder accurate analysis. I recommend submitting versions with enhanced sharpness and appropriate DPI (minimum 300) for publication.

5. The manuscript contains several grammatical issues that require attention. Notably, the Methods section should consistently employ the past tense. Additionally, there are instances of omitted words throughout the text, which affect clarity.

6. The table formatting does not conform to standard academic conventions. Specifically: (1) the tables should be presented in three-line format (top, bottom, and below header), and (2) table captions must be positioned above the tables rather than below. Please revise all tables to comply with these standard formatting requirements.

7. The manuscript contains inconsistencies in PIR categorization (text: <1.3/1.3–3.5/>3.5 vs. Table 1: <1.5/1.5–3.5/>3.5) and lacks justification for the chosen thresholds. Since PIR is a key covariate, its definition, rationale, and alignment with literature standards must be clarified in the Methods section.

8. The presentation of dietary data as mean ± SD requires demonstration of normal distribution, yet no normality tests are reported. Additionally, inconsistent units between text and tables need reconciliation. Both issues should be resolved to meet statistical reporting standards.

9. The manuscript incorrectly cites 'nhanesR' as the R package used for analysis; the correct package name is 'RNHANES.' This error must be corrected, and the authors should confirm that all analyses were conducted using the proper package to ensure methodological accuracy.

10. The use of log1.3(fold change) for differential expression analysis deviates from the conventional log2 approach. The authors must: (1) provide compelling justification for this methodological choice, and (2) demonstrate that results are robust to this alternative parameterization. Without such validation, the biological interpretation of differentially expressed genes remains questionable.

11. The analysis of total dietary flavonoids (29 compounds) and insulin resistance requires specialized methods for exposure mixtures. The employed weighted generalized linear models are unsuitable for this purpose, as they cannot properly handle the complex correlations and potential interactions among flavonoid subclasses. The authors should implement mixture-specific approaches (e.g., quantile g-computation) and revise their analyses accordingly.

12. The transition from epidemiological findings to experimental validation requires clearer justification. Given that multiple compounds showed significant associations with insulin resistance in the epidemiological analysis, the exclusive focus on anthocyanidins and hesperidin for in vitro studies needs explicit rationale to avoid potential selection bias. The authors should either justify this selective approach or expand their experimental validation.

13. Substantial inconsistencies exist in the reported number of compounds throughout the molecular docking section, with unexplained variations between different manuscript sections. The authors must: (1) reconcile these discrepancies, (2) document all compound exclusions with justification, and (3) provide a complete final compound list to ensure methodological rigor.

14. The target identification methodology requires revision, as pooling targets from all compounds obscures compound-specific mechanisms. The authors should: (1) analyze each compound's targets separately, (2) incorporate binding affinity data, and (3) apply proper statistical methods rather than simple frequency counts to identify biologically relevant key genes.

**To Reviewer #2**

1.We sincerely thank the reviewers for pointing out the format issues. To solve these problems, we carefully revised the entire manuscript. Specifically, all Chinese characters that appeared unintentionally have been identified and removed, and at the same time, inconsistencies and excessive spacing throughout the text have been thoroughly corrected. We apologize for the negligence at the time of initial submission and confirm that the manuscript has been carefully proofread to ensure consistent and correct format.

2.Thank you very much to the reviewers for pointing out the core methodological issues. We sincerely accept the defect you pointed out. In the initial analysis, although the use of traditional quartile grouping was a common statistical method, it was indeed inappropriate in the specific context of this study. We are deeply aware of the severity of this defect and have comprehensively revised the analytical methods to ensure that the results reflect the true biological effects. The following detailed modification plan:

(1) First of all, we carefully examine the concentration of the target compounds in the entire study population distribution characteristics, and based on the effect of biological significance, known threshold inflection point, concentration distribution, and, most importantly, ensure grouping can capture significant concentration gradient change, we define the boundary between the specific concentration range as a group. The extremely low concentration group was significantly lower than the daily required intake, the low concentration group was lower than the peak distribution of the population, the medium concentration group covered the main dietary intake range, and the high concentration group was slightly higher than the regular intake. The specific groupings are as follows:

Sum of all 29 flavonoids：[0,50]mg, (50,200]mg, (200,500]mg, >500mg

Anthocyanidins：[0,0.15]mg, (0.15,2.36]mg, (2.36,13.6]mg, >13.6mg

Isoflavones：[0,0]mg, (0.001,0.015]mg, (0.02,0.1]mg, >0.1mg

Flavan_3_ols: [0,5.3]mg, (5.03,17]mg, (17,170] mg, >170mg

Flavanones: [0,0.1]mg, (0.1,1]mg, (1,20]mg, >20mg

Flavones: [0,0.2]mg, (0.2,0.6]mg, (0.6,1.2]mg, >1.2mg

Flavonols: [0,8]mg, (8,14]mg, (14,24]mg, >24mg

(2) Secondly, we reanalyzed all relevant data using the newly defined grouping based on concentration gradients. Weighted multivariate regression analysis was adopted. With the low-concentration group as the reference, the β values between groups were compared, and the P for trend between groups was increased. Please refer to Table 2 for details.
(3) Finally, we detailed the new grouping strategy in the revised draft (lines 268-274). All tables and charts present the results using the new concentration gradient grouping. At the same time, the specific concentration range corresponding to each group is clearly marked in the legend. We would like to express our gratitude once again to the reviewers for their profound insights. The new method has significantly enhanced the biological rationality of the analysis.

3.Dear reviewer, thank you for the important questions you raised. The dietary flavonoid intake data in this study was derived from the flavonoid value database released by the United States Department of Agriculture. It should be noted that this database only includes dietary flavonoid intake data for the periods of 2007-2010 and 2017-2018, and does not include data from intermediate years such as 2011-2012, 2013-2014, and 2015-2016. As far as we know, all published flavonoid studies based on NHANES data have used data within the same year range. Therefore, unfortunately, we are unable to include the intermediate years in this analysis. Thank you for your understanding.

4.Thank you very much for your meticulous and rigorous review of the manuscript. Your opinions on the resolution of the chart are extremely crucial. We fully agree with the importance of high-quality images in ensuring the accuracy and readability of research results. We have resubmitted Figures 1 and 2 to ensure that their clarity, detail presentation and resolution (≥ 300 DPI) all meet the requirements of the journal.

5. Thank you very much to the reviewers for emphasizing these language issues. We strictly resolved all grammar issues through two steps: Firstly, the entire manuscript was professionally edited by native English-speaking researchers and scientifically written with professional knowledge. Meanwhile, we specifically rechecked the consistency of tenses in the method section and conducted a full-text screening for the omitted words. All program descriptions now uniformly use the past tense (for example, "samples were analyzed" instead of "samples are analyzed"). Secondly, restore missing articles or prepositions (for example, "the", "of", "to") to ensure syntactic integrity. Finally, the complex sentences in the article were reorganized to enhance clarity. These revisions have enhanced the readability and professionalism of the manuscript. We confirm that all the modifications have been recorded in the revised draft.
6. Thank you for your valuable suggestions. We have strictly followed academic norms to modify the full text table: Firstly, it has been uniformly changed to a three-line table format. At the same time, adjust the title positions and move all titles to the top of the table. Finally, ensure that the modified table fully complies with the requirements of the journal and that no original data has been altered.
7. Thank you very much for your detailed review of the article. We carefully reviewed relevant literature and, based on the poverty threshold set by the US Census Bureau (based on annual variations in family size and living costs) and the published NHANES article, we believe that low income (PIR < 1.3), middle income (PIR 1.3-3.5), and high income (PIR > 3.5). That is to say, the expression in the text is correct. Regarding the issue of inconsistent PIR classification in the chart, we have confirmed that the difference stems from an error in the data analysis stage. To ensure the robustness of the results, we have made the following revisions:
(1) In the method section, supplement the definition, basic principles and reasons for the classification basis of PIR, and add relevant references for supplementary explanation. (lines 130-137)
(2) In the method section, clearly define the PIR classification criteria, namely low (PIR<1.3), medium (1.3-3.5), and high (PIR>3.5). Meanwhile, unify the PIR classification in all texts to <1.3/1.3-3.5/>3.5 (including methods, results, and table notes).
(3) Reclassify the PIR data in Table 1 according to the above criteria. The modified data is as follows:the PIR data in table 1 reclassified according to the above standards, the modified data is as follows:

| **Variable** | **Total (n=3564)** | **Very low**  **(n=1458)** | **Low**  **(n=1115)** | **Moderate**  **(n=568)** | **High**  **(n=423)** | **P-value** |
| --- | --- | --- | --- | --- | --- | --- |
| **PIR, n (%)** |  |  |  |  |  | 0.001 |
| <1.3 | 940(16.77) | 466(21.35) | 274(14.39) | 121(15.10) | 79(11.28) |  |
| 1.3-3.5 | 1351(34.75) | 573(36.40) | 413(32.81) | 216(36.86) | 149(32.10) |  |
| >3.5 | 1273(48.48) | 419(42.25) | 428(52.80) | 231(48.04) | 195(56.62) |  |

(4) All subsequent statistical analyses were conducted based on correct standards.
8. We sincerely appreciate your valuable opinions. In response to your comments on the dietary flavonoid intake data, we acknowledge that, given the typical skewed distribution of such data, using the mean ± standard deviation is inappropriate. Therefore, we have made the following revisions to the entire manuscript: Firstly, in the methods section (Section 2.5), we have modified the statistical description of continuous variables: "If the continuous variable conforms to a normal distribution, it is expressed as mean ± standard deviation (SD) and a t-test is used." If it is a skewed distribution, it is expressed as the median (25th and 75th percentiles) [M(P25, P75)], and the Shapiro-Wilk test is used. This clarifies the criteria for choosing appropriate descriptive statistics and explicitly mentions the normality test used. Secondly, we changed all dietary flavonoid data in the full text from mean ± standard deviation to median (P25, P75), including the results section (Section 3.1) and Table 1. Finally, we unified the unit of dietary flavonoid intake throughout the entire manuscript to mg/d. This includes updates to the abstract, the results section (3.1), Tables 1, 2, 3, and any related legends. These revisions ensure stricter statistics and consistency when reporting our findings. Thank you for your incisive comments, which have made the article more perfect.
9. We sincerely apologize for the error in citing the R package name in the manuscript. Please understand that this was caused by our writing error. In fact, the software package used throughout the analysis process in this study was RNHANES (not "nhanesR"). We confirm that the following measures have been taken: Firstly, the R package name in the method section has been updated to "RNHANES" (line 174). Secondly, we revalidated all the analysis codes and confirmed that each statistical process was executed using the correct RNHANES package. Crucially, this correction is a strict terminology correction that does not affect any analytical output or scientific conclusion, as actual calculations are always carried out using valid software packages.
10. Your pursuit of academia commands our admiration. Thank you again for your hard work. The reason for choosing this method is that this public database is transcriptome sequencing of adipose tissue. Therefore, considering the characteristic that the mRNA expression difference in adipose tissue is not significant, we have reduced the multiple of difference. As for our research content - insulin resistance, one point that is different from other diseases is Even a 20-30% change in the expression levels of related genes (such as PIK3CG and MMP9) (i.e., 1.2-1.3 times) may lead to significant alterations in pathway function and seriously affect biological functions. The log1.3 threshold is more capable of capturing these moderate but biologically significant changes than the traditional log2 threshold. This is not our original creation but is supported by previous studies such as PMID: 33275239, 40517339, etc. For the data results, we supplemented Table S2 and figure S4 to present the results obtained by the log1.3 and log2 methods. The results showed that the key target gene PIK3CG/MMP9 was detected at different thresholds, while the GO/KEGG enrichment mode remained highly consistent among different thresholds.
11. Dear reviewer, we sincerely appreciate your valuable suggestions. The analytical approach of quantile g-computation (QGC) can indeed provide deeper insights for the study of exposure mixtures. However, after repeated deliberations by the team and consultations with statistical experts, we regretfully found that this method could not be implemented under the current research conditions. We sincerely apologize for this and earnestly ask you to understand the following restrictions:
(1) NHANES database limitations: The flavonoid data comes from a 24-hour dietary review, which inherently has recall bias. Moreover, 29 compounds are highly correlated (for instance, oranges provide both flavanones and citrus flavonoids), making it difficult to meet the independent exposure requirements of QGC.
(2) Methodological compatibility issues: Firstly, the complex sampling weights of NHANES fundamentally conflict with the quantile transformation algorithm of QGC. Secondly, the main toolkits used for analyzing QGC (such as qgcomp) do not support the integration of survey weights, and forced application will lead to bias.
(3) There is a strong correlation among the subclasses of flavonoids. Under the strong correlation, QGC will overestimate the effect of the high-exposure group.

Although QGC could not be implemented, we added an interaction test to verify whether the flavonoid subclasses had an impact on the relationship between total flavonoid intake and insulin resistance. The results are shown in the new Appendix S3. It is hoped that the reliability of the conclusion can be guaranteed to the greatest extent through this method. At the same time, we add the following limitations in the discussion section: "The analysis of the relationship between total flavonoids and insulin resistance in this study was limited by the completeness of exposure measurements in the NHANES database, and the complex correlations and potential interactions among various subcategories could not be fully analyzed." In the future, it is necessary to integrate multi-center cohort data and apply dedicated methods for exposure mixtures to deeply explore the synergistic/antagonistic mechanisms of flavonoid subclasses. (Lines 581-586)

We once again sincerely apologize for the methodological limitations. Your rigorous opinions have greatly enhanced the quality of this research, and we will continue to improve our relevant methodological capabilities.

12. We sincerely appreciate the reviewer's valuable suggestions, which have significantly improved the quality of our manuscript. To minimize potential selection bias and ensure comprehensive evaluation, we performed the following experimental validations: (1) Quantitative assessment of PIK3CG mRNA expression levels in 3T3-L1-derived adipocytes using RT-qPCR analysis following treatment with five distinct dietary flavonoids; (2) Systematic measurement of TNF-α secretion levels in cell culture supernatants employing ELISA methodology.
13. Once again, I pay tribute to your academic rigor. There is an inconsistency in the number of compounds between Section 2.6.3 and Section 2.6.5. The reason is the absence of some compound targets. We have supplemented the relevant explanations in Section 2.6.3 and sorted out the complete final compound list (Table S4).
14. We greatly appreciate the reviewer's insightful suggestions regarding our target identification methodology. As shown in Figure 5A, we have systematically analyzed the 12 flavonoids included in our network pharmacology study according to their respective subclasses.

To provide a more comprehensive evaluation, we performed standardized analyses incorporating binding affinity data through a weighted scoring system that considers three key parameters: 1) binding strength (normalized scores), 2) target frequency (number of compounds binding to each target), and 3) category coverage (number of flavonoid subclasses interacting with each target). The weighted total score was calculated as: Σ(normalized score) × target frequency × category coverage.

Our analysis revealed that PIK3CG scored significantly higher (290.4) than other targets (SYK 27.6, MMP9 26.1, PRKCB 0.41). This predominance stems from its broad-spectrum binding characteristics (10/11 compounds, covering all 4 subclasses). These methodological improvements and corresponding results have been incorporated into sections 2.6 (Methods) and 3.8 (Results) of our revised manuscript.

We believe this enhanced analytical approach provides a more rigorous and quantitative assessment of target importance while maintaining the integrity of our network pharmacology findings.

**Editorial Formatting Comments**

1. Please ensure that your manuscript meets PLOS ONE's style requirements, including those for file naming. The PLOS ONE style templates can be found at

<https://journals.plos.org/plosone/s/file?id=wjVg/PLOSOne_formatting_sample_main_body.pdf> and

[https://journals.plos.org/plosone/s/file?id=ba62/PLOSOne_formatting_sample_title_authors_affiliations.pdf](https://track.editorialmanager.com/CL0/https://journals.plos.org/plosone/s/file?id=ba62/PLOSOne_formatting_sample_title_authors_affiliations.pdf/1/010f0197eb667e5d-40bd06cb-bf93-4851-9d2a-90a2f0e9c580-000000/7ZHwUom9knd15URDy5RKfu2kIVRifpRttbpGoiwo2H8=219)

2. Please amend the manuscript submission data (via Edit Submission) to include author Zuocheng Wang.
3. Your ethics statement should only appear in the Methods section of your manuscript. If your ethics statement is written in any section besides the Methods, please delete it from any other section.
4. Please upload a new copy of Figure 3 as the detail is not clear. Please follow the link for more information:

<https://blogs.plos.org/plos/2019/06/looking-good-tips-for-creating-your-plos-figures-graphics/>

**To the editor**

1. We have revised the format of the manuscript to meet the style requirements of PLOS ONE as requested.
2. We have added Zuocheng Wang as an author to the submission system.
3. We have removed the ethics statement outside the Methods section.
4. We have modified Figure 3 and rendered Figure 3 and 4.
