# Supplementary material for: Dietary flavonoids may improve insulin resistance: NHANES, network pharmacological analyses and in vitro experiments
Source: PLoS One. 2025 Dec 5;20(12):e0338100. doi: 10.1371/journal.pone.0338100 (PMC12680246; doi:10.1371/journal.pone.0338100)
Supplement: Attachment — Submitted filename: Response_to_Reviewers_auresp_2.docx [file pone.0338100.s013.docx]

Dear editor of the *PLOS ONE*,

We are submitting our revised manuscript entitled “Dietary flavonoids may improve insulin resistance: NHANES, network pharmacological analyses and in vitro experiments” to the *PLOS ONE* for your reconsideration of its suitability for publication. All authors have read and approved the manuscript. We have carefully taken the reviewers’ comments into account and provided responses to each of the points raised by the reviewers. Please refer to the present manuscript text.

**Reviewer #1**: The revised manuscript has been improved, but there are some aspects that should be checked.
I agree with the authors that polyphenols can act as “drug-like” substances, but they are natural compounds, not drugs. Please, consider and revise through the manuscript.
Other points:


- Abstract: line 26, as a salt of cyanidin has been used, as explained to this referee, please, state this clearly in the abstract.

- Material and methods: Section 2.7: Please, state clearly that the cells were incubated with cyanidin, as was done for hesperidin. Also, include the explanation given to this referee for selecting cyanidin as a representative anthocyanidin compound.
Please, note that RT-qPCR is not included in this section as well as the statistical analysis performed in cell experiments.

- Results: Section 3.1: Please, comment about HDL, TG, insulin levels, all other parameters included in table 1 that have been not described by the authors.
Section 3.9: Please, state clearly that the cells were incubated with cyanidin. Please, note that anthocyanidins constitutes a wide group of compounds and that using this term is confusing, or may even refer to a mixture of compounds, which is not the case, as explained to this referee.
Please, explain why three additional polyphenols have been included. Which was the rationale for the selection? Why each compound was tested at a different concentration? Please, explain.

- Tables: Please, consider using abbreviations for well-known parameters (HDL; TG, etc) in table 1.

- Figures: In figure 7, please, replace anthocyanidin with cyanidin, the compound used for the cellular studies.


There are still certain grammatical and typo errors through the manuscript that require attention.

**Response to reviewer #1**

1. Thank you very much for your suggestion. We have replaced all the words related to "drug" or related to treatment in the entire text, making the research closer to the theme of natural medicines and diets rather than drugs.
2. We have added relevant explanations in the methods section of the abstract.
3. We made amendments in 2.7, clearly stating that the cells were incubated with cyanidin. We provided a brief explanation for the reasons behind choosing anthocyanins as the representative, and added the missing RT-qPCR and the statistical analysis conducted in cell experiments.
4. Thank you for your valuable suggestions. We have made supplements and revisions in Section 3.1 of the Results section based on your suggestions. Specifically, we have added descriptions of HDL, TG, fasting insulin levels, and fasting blood glucose levels. For the revised content, please refer to Section 3.1 of the manuscript.
5. We thank the reviewer for this insightful question, which allows us to clarify the strategic expansion of our experimental validation. We have corrected the ambiguous terms in the paragraph.

The inclusion of the three additional flavonoids (kaempferol, luteolin, and myricetin) was to validate the generalizability of our network pharmacology predictions, demonstrating that the amelioration of insulin resistance is a shared property across multiple flavonoid subclasses, not limited to the initial candidates. The use of different concentrations for each compound was based on prior CCK-8 cytotoxicity assays, where we selected the highest non-cytotoxic dose (below IC10) for each to ensure that the observed improvements in glucose uptake and gene expression were due to genuine bioactivity rather than compromised cell viability. This approach ensured both the biological relevance and the experimental rigor of our functional validation.

6. Following your suggestions, we have revised Table 1. Standard abbreviations were used for parameters such as physical activity (PA), coronary heart disease (CHD), high density lipoprotein (HDL), triglycerides (TG), and fasting insulin (FINS). And in the annotation section of the table, all abbreviations are fully explained to ensure their clarity.
7. We have corrected the incorrect words in Figure 7.

**Reviewer #2:** I have reviewed the authors' revised manuscript. In the process of revising the manuscript, the authors made revisions based on my suggestions and made responses to all the questions I raised. Therefore, I suggest that the journal accept this article.

**Response to reviewer #2**

Thanks for your hard work.
