# Supplementary material for: Dietary flavonoids may improve insulin resistance: NHANES, network pharmacological analyses and in vitro experiments
Source: PLoS One. 2025 Dec 5;20(12):e0338100. doi: 10.1371/journal.pone.0338100 (PMC12680246; doi:10.1371/journal.pone.0338100)
Supplement: Attachment — Submitted filename: Response_to_Reviewers_auresp_3.docx [file pone.0338100.s014.docx]

Dear editor of the *PLOS ONE*,

We are submitting our revised manuscript entitled “Dietary flavonoids may improve insulin resistance: NHANES, network pharmacological analyses and in vitro experiments” to the *PLOS ONE* for your reconsideration of its suitability for publication. All authors have read and approved the manuscript. We have carefully taken the reviewers’ comments into account and provided responses to each of the points raised by the reviewers. Please refer to the present manuscript text.

**Reviewer #1**: The revised manuscript has been improved, and just minor aspects should be checked.

- Material and methods: Section 2.7: Please, state clearly the number of cells seeded to perform each assay.

For the RT-qPCR, please, also include the micrograms of RNA transcribed and the nanograms of RNA amplified.

There are still certain grammatical and typo errors through the manuscript that require attention.

**Response to reviewer #1**

1. Thank you very much for your suggestion. We have already listed in Section 2.7 the number of cells used in the experiment, as well as the micrograms of transcribed RNA and the nanograms of amplified RNA.
2. We have once again revised the inappropriate terms such as "hypertension" throughout the entire text.
